# Dynamics of the Transcriptome during Human Spermatogenesis: Predicting the Potential Key Genes Regulating Male Gametes Generation

**DOI:** 10.1038/srep19069

**Published:** 2016-01-12

**Authors:** Zijue Zhu, Chong Li, Shi Yang, Ruhui Tian, Junlong Wang, Qingqing Yuan, Hui Dong, Zuping He, Shengyue Wang, Zheng Li

**Affiliations:** 1 Department of Urology, Ren Ji Hospital, School of Medicine, Shanghai Jiao Tong University, 1630 Dongfang Road, Shanghai 200127, China; 2 Department of Andrology, Urologic Medical Center, Shanghai General Hospital, Shanghai Jiao Tong University, 100 Haining Road, Shanghai 200080, China; 3 Shanghai-MOST Key Laboratory of Health and Disease Genomics, Chinese National Human Genome Center at Shanghai, 250 Bibo Road, Shanghai 201203, China; 4 Clinical Stem Cell Research Center, Ren Ji Hospital, School of Medicine, Shanghai Jiao Tong University, 1630 Dongfang Road, Shanghai 200127, China; 5 Shanghai Key Laboratory of Reproductive Medicine, Shanghai, 200025, China

## Abstract

Many infertile men are the victims of spermatogenesis disorder. However, conventional clinical test could not provide efficient information on the causes of spermatogenesis disorder and guide the doctor how to treat it. More effective diagnosis and treating methods could be developed if the key genes that regulate spermatogenesis were determined. Many works have been done on animal models, while there are few works on human beings due to the limited sample resources. In current work, testis tissues were obtained from 27 patients with obstructive azoospermia via surgery. The combination of Fluorescence Activated Cell Sorting and Magnetic Activated Cell Sorting was chosen as the efficient method to sort typical germ cells during spermatogenesis. RNA Sequencing was carried out to screen the change of transcriptomic profile of the germ cells during spermatogenesis. Differential expressed genes were clustered according to their expression patterns. Gene Ontology annotation, pathway analysis, and Gene Set Enrichment Analysis were carried out on genes with specific expression patterns and the potential key genes such as *HOX*s, *JUN*, *SP1*, and *TCF3* which were involved in the regulation of spermatogenesis, with the potential value serve as molecular tools for clinical purpose, were predicted.

It was reported that about 10%–15% couples suffering from infertility in which 50% of the cases were caused by male factors^1^,^2^. Spermatogenesis disorder was one of the main causes of male infertility while key genes which could serve as molecular tools for the diagnosis and treatment of spermatogenesis disorder remained to be identified.

Using the rodent models, hundreds of gene defects had been associated with abnormal spermatogenesis^3^,^4^, and with the help of Gene Array, the dynamic of rodent transcriptional profile during spermatogenesis had been revealed^5^,^6^. Specific stages of gene expression in mouse spermatogenesis had been profiled. Based on a construction and validation of a comprehensive subtractive cDNA microarray, the comparison of the testicular transcriptome between normal and infertile mice helped us to depict the molecular mechanism of spermatogenesis and the possible pathology of infertility^7^.

However, the course of human male gamete production is somewhat different from that of rodent and the finding on rodent is not essentially identical to that of human beings. For example, the functions of some Y-chromosome conserved genes in mouse spermatogenesis were different from that in human spermatogenesis. Deletion of most mouse *Rbm* genes only caused some sperm dysmorphology while on human, *RBM* was expressed during meiosis and deletion of *RBM* lead to meiosis arrest^8^,^9^. Mouse *Dby* was not essential for pre-meiosis spermatogenesis while, on human, its homology was mainly expressed in spermatogonia^10^. These facts indicated that fundamental differences existed in the biology of human germ cell and the necessary of researches on the transcriptome of human germ cell directly.

Up to now, there were only a few gene defects were identified to be related to human infertility. The causes of many infertile diseases were not clear yet. It was difficult for doctors to provide effective treatments for these infertile patients. Besides, we did not even know the basic molecular mechanism of human spermatogenesis. The determination of the dynamic of transcriptional profile during human spermatogenesis would facilitate our understanding of the molecular drive of human male gamete production, as well as the root cause of male spermatogenesis dysfunction.

In another hand, with the progress in the research on cell plasticity, it became possible to modulate cell features via regulating the expression of some key genes. If we identified the key genes that regulate the process of spermatogenesis, we could make use of them to modulate the cell, promoting the generation of male gamete, which would give hope to those who suffering from spermatogenesis failure.

## Results

### Cell sorting and verification of sorted cells

**Figure 1:**
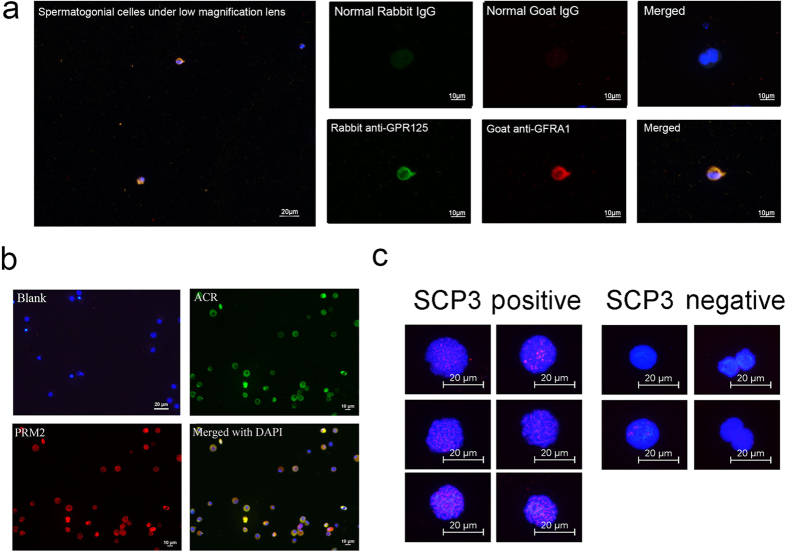
The identification of sorted germ cells. Germ cells of different differentiated stages were sorted via FACS and MACS, and immune staining were applied to identification the sorted cells. (**a**) Immunofluorescence identified MACS sorted CD90+ cells. The figure on the left shows cells observed under low magnification lens. The quantity of sorted CD90+ cells was very low. The figures on the right show the detailed staining results. About 90% of these cells were GFRA1 and GPR125 positive, suggested that these cells were enriched undifferentiated spermatogonias. Blank was provided by staining with normal IgGs; (**b**) Immunofluorescence identified FACS sorted haploid cells. Over 85% of these cells were PRM2 and ACR positive, suggested that these cells were enriched spermatids. Blank was provided by staining with normal IgGs; (**c**) Spread identified FACS sorted tetraploid cells. Over 200 cells were counted. The figure shows the typical positive cells and negative cells. Over 80% of these cells were definitely SCP3 positive, suggested that these cells were enriched primary spermatocytes.

Testis tissues were obtained from 27 patients with obstructive azoospermia (OA) in which case the spermatogenesis was thought to be normal via surgery. The combination of Fluorescence Activated Cell Sorting (FACS) and Magnetic Activated Cell Sorting (MACS) were used to sort germ cells from testicular biopsy. Immonuflourescence and meiosis spread were performed to identify the sorted cells, including haploid cells, tetraploid cells and CD90+ diploid cells which were supposed to be enriched spermatid, primary spermatocyte and undifferentiated spermatogonias, respectively. It was confirmed that the morphology of these cells were identical to spermatid, spermatocyte and undifferentiated spermatogonias (Fig. S1). For haploid cells and tetraploid cells, at least 200 cells were counted for the calculating of positive ratio. For CD90+ cells, due to the low density of the cell, we count the cells we could observe as many as possible. About 90% CD90+ cells were GPR125 and GFRA1 positive (Fig. 1a). While over 85% haploid cells were PRM2 and ACR positive (Fig. 1b). Meiosis spread showed that 80% of the sorted tetraploid cells were SCP3 positive (Fig. 1c).

### Profiling of RNA transcription in human male germ cells during spermatogenesis

**Figure 2:**
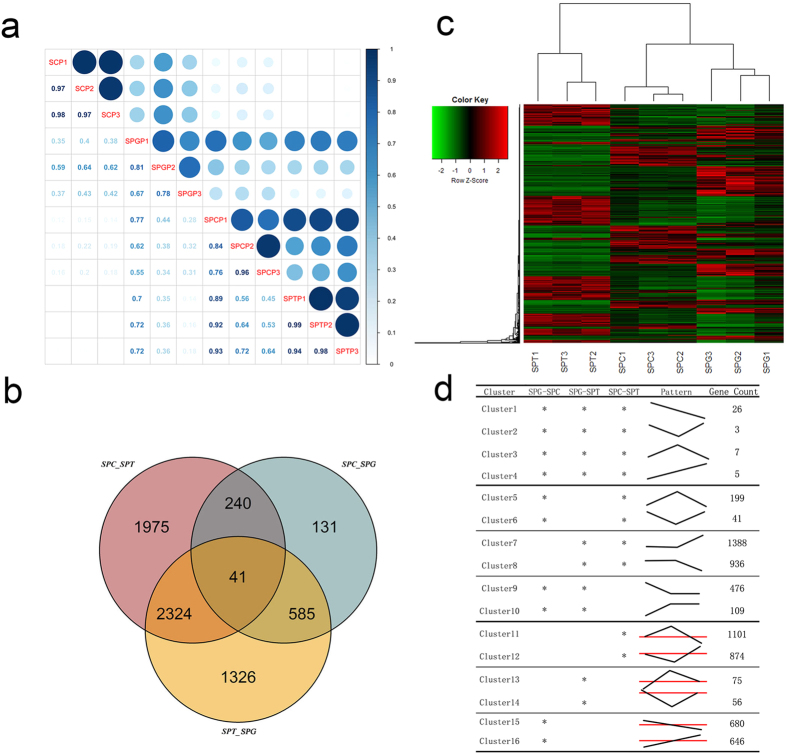
The differential expression of genes during spermatogenesis. The data obtained via RNA-Seq revealed the differential expression profile among the three typical germ cells. (**a**) The relevance of the expression profiles among Sertoli cells (SC), undifferentiated spermatogonias (SPG), primary spermatocytes (SPC), and spermatids (SPT) was showed. Three biological repeats were performed on each type of cells, annotated with P1, P2, and P3 respectively. The correlation coefficients between two groups of cells were noted, and the size and depth of the dots also represented the relevance between the results. This figure clearly showed the repeatability of the sequencing and the difference among cells from different groups; (**b**) Venn Diagram showed the number of differential expressed genes in SPG, SPC, and SPT. The left cycle represented the differential expressed genes between SPC and SPT; the right cycle represented the differential expressed genes between SPC and SPG; the lower cycle represented the differential expressed genes between SPT and SPG. Numbers labeled in each part show the amount of the genes with the corresponding expression patterns; (**c**) Heatmap consisted of differential expressed genes during spermatogenesis. A total of 6, 622 differential expressed genes were included. Three biological replicates were performed on each type of cells; (**d**) Genes were clustered according to their expression patterns. Asterisk “*” showed where significant difference existed. The curve briefly showed approximate expression trends of the genes in each clusters.

RNA-Seq (RNA Sequencing) was employed to profile the RNA transcription in human male germ cells during spermatogenesis. A total of 24,877 distinct transcripts were profiled in the cells. The results derived from the same type of cells were highly consistent (Fig. 2a,b).

The differentially expressed genes among them were analyzed. Only the genes that with a basemean >200, change fold >3, and padj <0.001 were list for a strict result. A total of 4,580 genes were identified to be differentially expressed between spermatocytes and spermatids with 2,311 genes up-regulated in spermatids and 2,269 up-regulated in spermatocytes. Atotal of 997 genes were identified to be differentially expressed between undifferentiated spermatogonias and spermatocytes with 602 genes were up-regulated in undifferntiated spermatogonias and 395 genes were up-regulated in spermatocytes. A total of 4,276 genes were identified to be differentially expressed between undifferentiated spermatogonias and spermatids with 2,123 genes were up-regulated in undifferentiated spermatogonias and 2,153 were up-regulated in spermatids. (Fig. 2c).

### Expression pattern

Differential expressed genes were clustered into different clusters according to their expression patterns. A total of 16 clusters were identified (Fig. 2d). Cluster 1–4 represented the genes that significantly differentially expressed in all three types of sorted cells. Among the 4 clusters, Cluster 1 represented the genes whose expressions were up-regulated continouselly during spermatogenesis; Cluster 2 represented the genes whose expressions were down-regulated in spermatocytes; Cluster 3 represented the genes whose expressions were up-regulated in spermatocytes; Cluster 4 represented the genes whose expressions were down-regulated continouselly during spermatogenesis. Cluster 5–10 represented the genes that were preferentially expressed or inhibited in specific cell. Cluster 5 represented the genes that were specific or preference expressed in spermatocytes; Cluster 6 represented the genes that were inhibited in spermatocytes; Cluster 7 represented the genes that were specific or preference expressed in spermatids; Cluster 8 represented the genes that were inhibited in spermatids; Cluster 9 represented the genes that were specific or preference expressed in spermatogonia,; Cluster 10 represented the genes that were inhibited in spermatogonia. The expression of the genes belonged to Cluster 11–16 were more complicated, the expression of these genes were significantly different between two types of sorted cells, while the differences were not significant when compared with the third type of sorted cell. The distinct expression pattern of these genes may indicate the roles they play during spermatogenesis.

### Quantitative PCR verification of sequencing data

**Figure 3:**
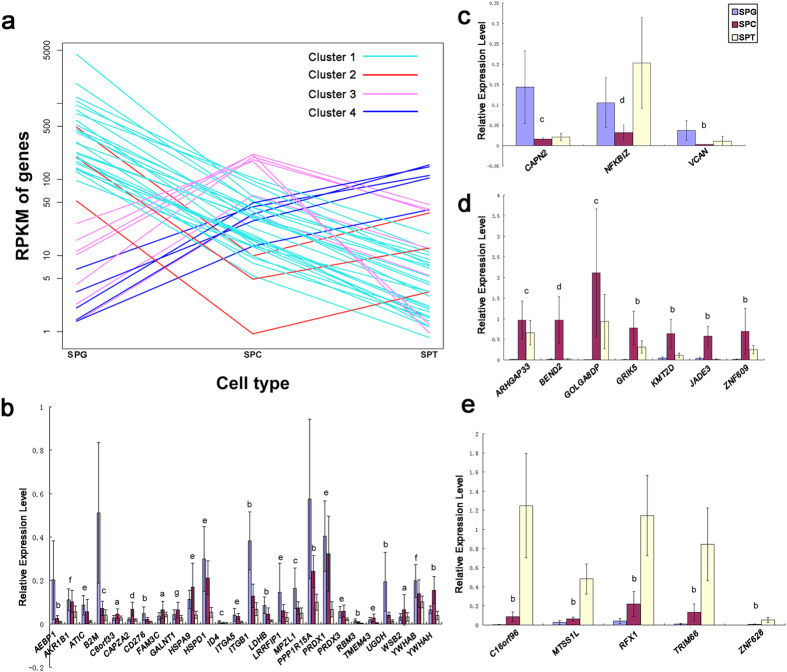
Q-PCR verification of RNA-Seq. A total of 41 genes from cluster 1–4 were selected to validate the results of RNA-Seq via Q-PCR. (**a**) The expression curves of the 41 selected genes; (**b**) Q-PCR results of genes from Cluster 1; (**c**) Q-PCR results of genes from Cluster 2; (**d**) Q-PCR results of genes from Cluster 3; (**e**) Q-PCR results of genes from Cluster 4. The overall expression trends of the 41 genes were consisted with the results of RNA-Seq, suggested that these results were reliable. (T-test was performed. The bars represent standard deviation of the samples, n = 7. Significant difference were determined when p-value < 0.05; Abbreviation: SPG = spermatogonial cell; SPC = spermatocyte; SPT = spermatid; a = no significant difference among the three populations; b = the three populations were significantly different from each other; c = there were significant differences between SPG and SPC, SPG and SPT, while no significant difference between SPC and SPT; d = there were significant differences between SPG and SPC, SPC and SPT, while no significant difference between SPG and SPT; e = there were significant differences between SPG and SPT, SPC and SPT, while no significant difference between SPG and SPC; f = there was significant difference between SPG and SPT, while no significant difference between SPG and SPC, SPC and SPT; g = there was significant difference between SPC and SPT, while no significant difference between SPG and SPC, SPG and SPT).

Real time quantitative PCR (Q-PCR) was performed to verification the results of sequencing. The 41 genes from Cluster 1–4 were selected to be detected (Fig. 3a shows their expression curves); *ACTB* was chosen as the internal reference. All selected genes had significant difference expression between spermatocytes and spermatids, which identical to that of sequencing. When compared with the expression level in undifferentiated spermatogonias, the general expression tendency was agree with that of sequencing, with only 9 genes out of 41 different from that of sequencing (Fig. 3b–e). Overall, these results supported that the date provided by RNA-Seq is reliable.

### Transcripts profile match cellular protein localization patterns

**Figure 4:**
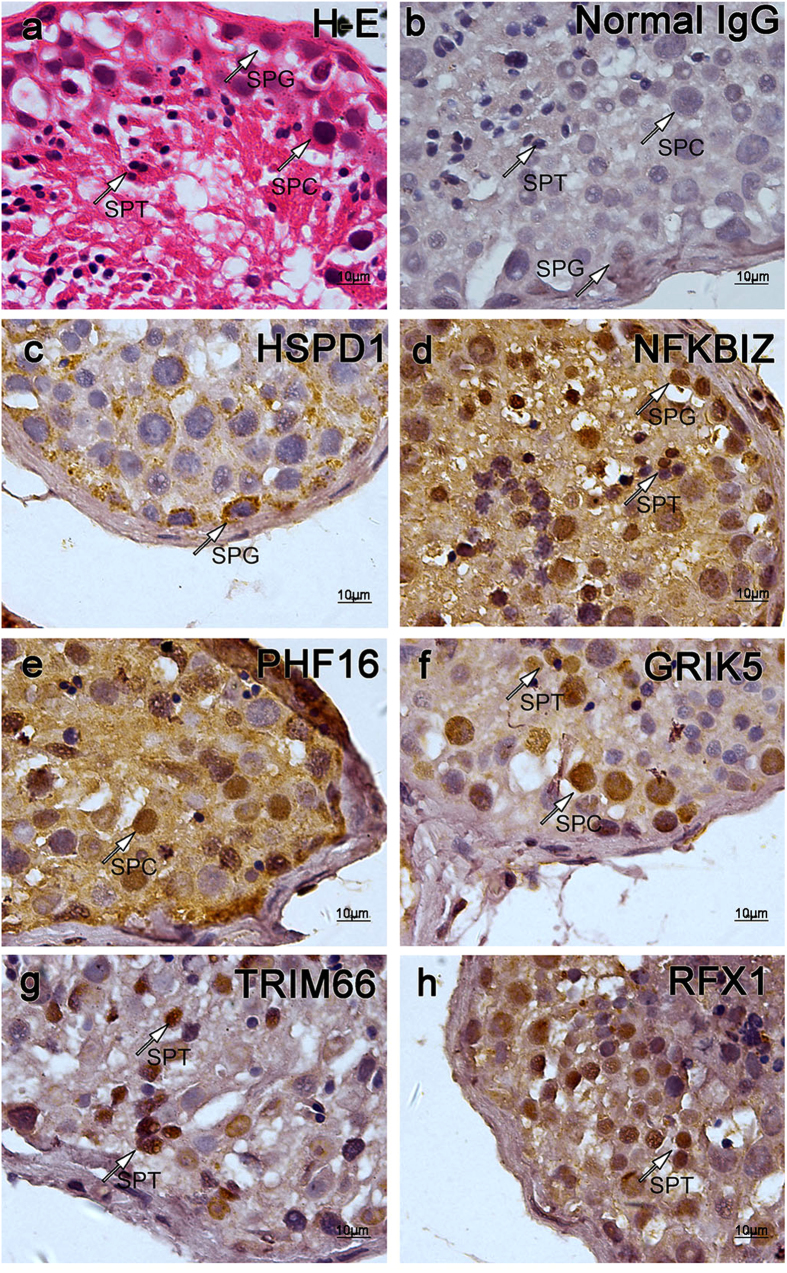
Immunohistochemistry certified the localization of the proteins of corresponding genes in testicular tissues. A total of 6 genes were selected to be detected on testicular tissue sections derived from OA patients with normal spermatogenesis via immunohistochemistry, normal Rabbit IgG was used as primary antibody for negative control. Typical germ cells were pointed by arrows. (**a**) Hematoxylin-eosin (H-E) staining of testicular tissue section. Normal spermatogenesis could be observed; (**b**) Negative control stained with normal IgG. Though there was a little non-specific background staining, no specific staining could be observed; (**c**) Section stained with anti-HSP60 (HSPD1) antibody. HSPD1 belongs to Cluster 1. (See Figs 2d and 3a) Spermatogonial Cells were stained in brown; (**d**) Section stained with anti-NFKBIZ antibody. NFKBIZ belongs to Cluster 2. Spermatogonial cells and some spermatids were stained in brown; (**e**) Section stained with anti-PHF16 (JADE3) antibody, PHF16 belongs to Cluster3. Spermatocytes were stained in brown; (**f**) Section stained with anti-GRIK5 antibody. GRIK5 belongs to Cluster 3. Many germ cells were stained in brown, among which spermatocytes and some spermatids were stained in a relatively dark color; (**g**) Section stained with anti-TRIM66 antibody. TRIM66 belongs to Cluster 4. Spermatids were stained in brown; (**h**) Section stained with anti-RFX1 antibody. RFX1 belongs to Cluster 4. Spermatids were stained in brown. These distribution patterns were mostly consisted to that of trancripts. (Abbreviation: SPG = spermatogonial cell; SPC = spermatocyte; SPT = spermatid.)

Immunohistochemistry was performed to detect the expression of genes *in situ*. A total of 6 genes with typical expression patterns were detected. These genes included *GRIK5*, *PHF16*, *RXF1*, *TRIM66*, *NFKBIZ*, and *HSPD1*. Among these proteins, HSPD1, whose corresponding transcript belongs to Cluster 1, is solely expressed in spermatogonial cells (Fig. 4c); NFKBIZ, whose corresponding transcript belongs to Cluster 2, is mainly expressed in spermatogonial cells and some spermatids (Fig. 4d); PHF16, whose corresponding transcript belongs to Cluster 3, is solely expressed in spermatocyte (Fig. 4e); GRIK5, whose corresponding transcript belongs to Cluster 3, is preferentially expressed in spermatocytes and some spermatids (Fig. 4f); RXF1 and TRIM66, whose corresponding transcripts belong to Cluster 4, are mainly expressed in spermatids (Fig. 4g,h); The localization patterns of the proteins of these genes were mostly matched the expression pattern profiled via sequencing.

### Gene Ontology (GO) analysis of differentially expressed genes

Blast2GO was used to make functional and GO annotation on differential expression genes. Genes of Cluster 5, 6, 7, 8, 9, and 10 were focused to be analysis due to the specificity of their expression (Fig. S2). From the annotation, some genes with specific functions could be screened. For example, in Cluster 5, a total of 44 genes were annotated with nucleic acid binding activity, among which, a total of 19 genes were annotated with transcription factor or cofactor activity. These genes are preferentially expressed in spermatocytes, which are supposed to be the regulator of meiosis. Interestingly, a total of 7 genes out of these genes belong to *HOX* family, indicating the important roles of *HOX* genes play in meiosis process.

### Pathway involved in spermatogenesis

Pathway analysis through KEGG (Kyoto Encyclopedia of Gens and Genomes) pathway mapping database provided the common pathway information involved in spermatogenesis. The detailed enriched pathway information was showed in supplementary table 1. The mostly common enriched pathway in all three cells is purine metabolism pathway. Pathways related with proliferation are the main pathways in spermatogonial cells. In spermatocytes, the mostly enriched pathways are progesterone-mediated oocyte maturation and oocyte meiosis. Pathways related with signaling transduction are the core pathways in spermatids. These enriched pathways may reflect the function features of these cells.

### Stage-specific enrichment of transcription factor targets during spermatogenesis

Gene Set Enrichment Analysis (GSEA) was performed to make enrichment analysis of the transcription factor targets of the genes differentially expressed during spermatogenesis. Corresponding transcription factors recognizing the most enriched motifs were listed in Table 1. In spermatogonial cells, the most influential transcription factors are NFATs, SP1, LEF1, TCF3, MAZ, JUN, PAX4, and MLLT7; in spermatocytes, the most influential transcription factors are SP1, TCF3, MAZ, LEF1, PAX4, MLLT7, MYOD1, TCF8, and NFAT; in spermatids, the most influential transcript factors are TCF3, SP1, LEF1, PAX4, MLLT7, MAZ, JUN, NFAT, and REPIN1. These transcription factors were putative key regulator of spermatoigenesis.

## Discussion

The diagnosis and the treatment of male infertility caused by spermatogenesis failure would be facilitated if we determine the dynamic of gene expression during the spermatogenesis and identify the key genes regulating the generation of gametes. To determine the dynamic of gene expression during spermatogeneis, it is crucial to divide germ cells from testicular tissues into different differentiated populations.

Since the time-point of the first spermatogentic wave of rodents had been determined^11^,^12^, it was possible to screen the RNA profiles of germ cells at different stage via sacrificing juvenile murine at different age^5^,^7^,^13^. Nevertheless, it is not suitable for research on human beings, and the results obtained from these works only reflected the abundance of genes in whole tissues, which may influenced by the abundance of the cells concerned. Hence, cell sorting is essential for transcriptome research.

To sort germ cells from testis tissues, some methods had been developed. Among these methods, STA-PUT velocity sedimentation was the most accepted method^14^,^15^,^16^,^17^. However, a large amount of tissue was required for STA-PUT which was difficult to gain from patients while the trancriptome research demanded sufficient samples with a high purity. There had been work on human beings used a elutriation rotor to isolate pachytene spermatocytes and spermatids for microarray analysis^18^. Yet, that work failed to collect enough sample for biological replications, and spermatogonial cells were not included.

### The combination of FACS and MACS is suitable for germ cells sorting from clinical biopsy

In current work, FACS associated ploidy sorting was used to isolate cells with different DNA ploid. CD90+ cells were sorted by MACS. After sorting, sorted cells were identified via detecting the markers of spermatid, spermatocyte and undifferentiated spermatogonias. Over 85% sorted haploid cells were PRM2 and ACR positive, indicating that the sorted haploid cells were enriched spermatid; About 90% sorted CD90+ cells were GPR125 and GFRA1 positive, indicating that the sorted CD90+ cells were enriched undifferentiated spermatogonias. Meiosis spread was performed to identified the sorted tetraploid cells, and it was determined that over 80% of the sorted tetraploid cells were SCP3 positive, indicating these cells were enriched primary spermatocytes. These results suggested that the combination of FACS and MACS could be an efficient method to sort germ cells from testicular biopsy into spermatid, primary spermatocyte and undifferentiated spermatogonias, and the sorted cells were pure enough to be used for sequencing purpose.

### The specificity of gene expression during spermatogenesis and its implication of gene function

Comparing the transcript profile of the three typical spermatogenic cell populations, it was noticed that some genes showed expression preference. It was interesting that the changes of these genes were coincided with the development process of germ cells. These differentially expressed genes were clustered according to their expression patterns. Genes from Cluster 5 are preferentially expressed in spermatocytes, indicating their potential roles in meiosis; Genes from Cluster 7 are preferentially expressed in spermatids, indicating their potential roles in spermiogenesis; Genes from Cluster 9 are preferentially expressed in spermatogonias, indicating their potential roles in proliferation.

Transcription factors, which serve as the switches for the regulation of downstream genes expression, playing a key role in biology events, were screened from these genes through GO analysis. Among these transcription factors, many were reported to have important regulatory functions during embryo development, tumorgenesis and neurons development.

**Figure 5:**
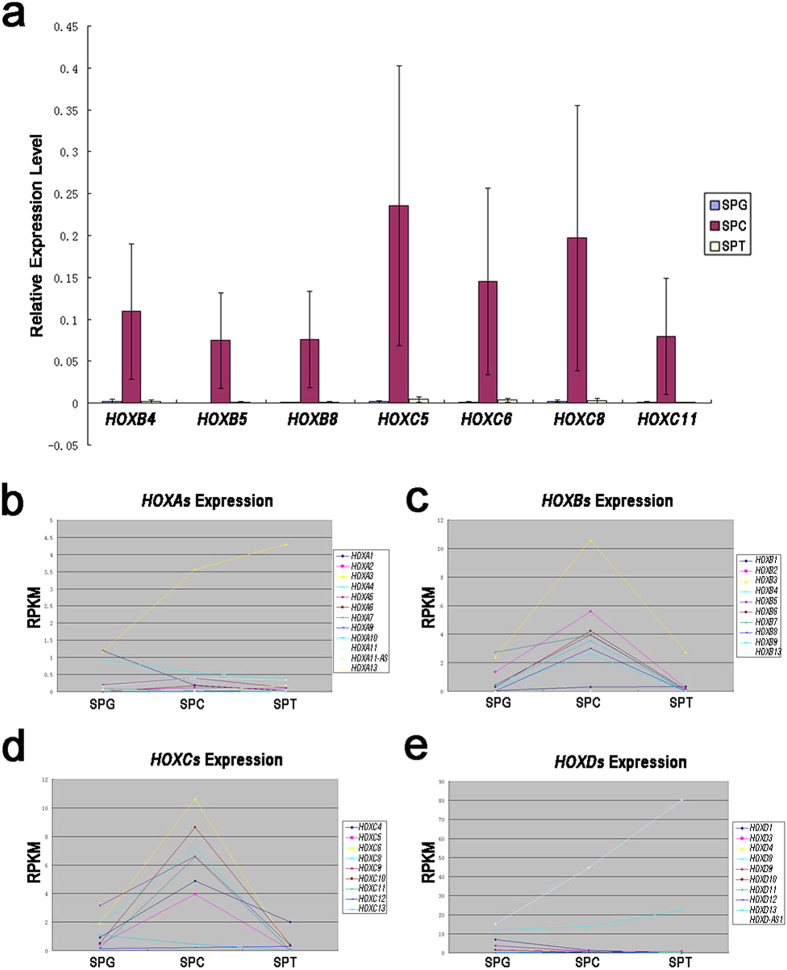
Specifically expression of *HOX* genes in spermatocytes. Q-PCR was performed to verification the expression pattern of *HOXB* and *HOXC* genes and mean RPKM values were used to generate curves of the expression patterns of *HOX* genes during spermatogenesis. (**a**) Q-PCR showed that selected *HOXB* and *HOXC* genes were specifically expressed in spermatocytes, suggesting these genes may play an important role in meiosis. (T-test was performed. The bars represent standard deviation of the samples, n = 7. Significant difference were determined when p < 0.05; Abbreviation: SPG = spermatogonial cell; SPC = spermatocyte; SPT = spermatid.); (**b**) Expression pattern of *HOXA* genes; (**c**) Expression pattern of *HOXB* genes; (**d**) Expression pattern of *HOXC* genes; E) Expression pattern of *HOXD* genes. These results suggested that *HOXB* and *HOXC* genes were specifically expressed in spermatocytes, while *HOXA* and *HOXD* genes lack of expression specificity. (Abbreviation: SPG = spermatogonial cell; SPC = spermatocyte; SPT = spermatid).

It was noticed that in current study, *HOX* genes, especially *HOXB*s and *HOXC*s, were specifically expressed in spermatocytes (Fig. 5a–e), which had not been reported in previous works. *HOX* genes were widely known to be crucial for limb development^19^; it was also reported that some members of *HOX* family are important for neuron development^20^. But it was never concerned what role these genes play during spermatogenesis. The specific expression of *HOX* genes in spermatocytes indicated that they could be a regulator for meiosis process. In recent research, it was reported that some *HOX* genes decide the polarity of cells, organizing the direction of spindle during cell division^21^, while spermatocytes which undergo meiosis would experience the re-organization of chromosome which should be regulated intensively. *HOX* genes may be involved in the accurate regulation of chromosome behavior during meiosis.

Besides the coding genes mentioned above, non-coding genes may also play important roles in spermatogenesis. Due to the methods employed to extract RNAs in our study, miRNAs were lost from our library. While long non-coding RNAs (lncRNAs) were remained and recorded. These RNAs included lincRNAs and some non-coding transcript varieties of coding genes. A total of 1,800 lncRNAs were recorded in our work, in which 157 were differential expressed during human spermatogenesis (Table 2). The functions of these non-coding RNAs have not been determined, but from the expression patterns of these non-coding RNAs, we could relate them with some process during spermatogenesis. For example, non-coding RNAs belong to cluster 11 may be important to meiosis because of their up-regulation in spermatocytes; non-coding RNAs belong to cluster 12 and 16 may be important to spermiogenesis because of their up-regulation in spermatids. Most of the differentially expressed non-coding RNAs belong to Cluster 8, which is down-regulated in spermtids. These RNAs may important for pre-meiotic development of germ cells, and no longer need for spermiogenesis. Of course, these speculations need to be confirm via further works.

### Fundamental differences existed in the biology of human germ cell from that of rodent

As mentioned previously, there had been some transcriptomic researches on spermatogenesis of rodent^5^,^6^,^7^, and Chalmel *et al.* performed two works on human^18^,^22^. The works of Chalmel improved our understanding of human spermatogenesis. However, one of their works used the tissue derived from patients with spermatogenesis disorder without sorting; another employed an elutriation rotor to sort the germ cells without efficient biological replicates and lack the data of spermatogonial cells. While in our work, a total of 15 individual samples were collected for sequencing and other 9 individual samples were prepared for the verification of sequencing results, made the results more reliable.

One of the works of Chalmel *et al.* compared the spermatogenesis between human and rodent^18^. The conservation between human and rodent was demonstrated. As a process that critical for the reproduction, the course of gametogenesis is essentially conserved. However, during the evolution, human also derived some distinguished features in spermatogenesis compared with that of rodent. For example, *DAZ* genes are essential for meiosis in human beings while they do not even exist in rodent^23^.

Schultz *et al.* screened the transcriptomic profile during spermatogenesis of mouse and clustered the differentially expressed genes according to their expression patterns just like we did^5^. Some typical genes of each cluster were listed, and a brief comparison of their expression patterns between human and mouse were made. A total of 25 genes whose homologues could be found in both human and mouse were selected for comparison. These genes included *TCAM1P*, *TEX101*, *ADAM2*, *ACR*, *ACRBP*, *CATSPER2*, *PGK2*, *LDHC*, *TCP10*, *TCP11*, *ADAM18*, *SPA17*, *SPAG5*, *TCTE3*, *TCTE1*, *TESK1*, *CATSPER1*, *GSG1*, *ODF1*, *PRM1*, *PEM3*, *TNP2, PLCZ1*, *TEX264*, and *GFRA1*. The expression patterns of 4 out of the 25 genes were different between human and mouse, including *TCAM1P*, *TEX101*, *ADAM2*, and *TCP10*. All of these genes were reported to be up-regulated in testis from 14 day postpartum at what time meiosis begins and maintained at a high level until sexually maturation in mouse. While our data showed that these genes were up-regulated in primary spermatocytes, and then down-regulated in spermatids (See Tab. S2). The up-regulation of these genes indicated that these genes were involved in meiosis in both species. In mouse, the maintaining of high level expression of these genes suggested that these genes may also play a role in spermiogenesis, while they may be not so crucial for spermiogenesis of human. Again, our work show that fundamental differences existed in the biology of human germ cell from that of rodent.

### The potentially key genes that modulate expression profile during spermatogenesis

With the progress in the research on cell reprogramming, the plasticity of cells became more and more attracting.

To artificially mould a cell, the straight way is to make the cell express all the genes needed to support the function of the target cell. However, regulating the expressions of all genes to achieve the molding purpose seems to be an impossible work.

Another way is to find the key factors that located at the hubs of the gene regulation network, regulating the expression of massive dowmstream genes.

As mentioned above, transcription factors could be considered to be the key switches regulating gene expressions. It has been proved that over-expression of some key transcription factors involved in the regulation of cell pluripotency could convert somatic cells into cells with embryo stem cell features^24^,^25^. Recently, it was even approved that fibroblast could transdifferentiate into hepatocyte via introducing some key transcription factors expressed in hepatocyte^26^,^27^.

RNA-binding proteins also showed their critical role in the regulation of cell plasticity. They play their role via post-transcription regulation^28^. It was reported that *DAZ* family genes, a family of RNA-binding proteins, could promoting embryonic stem cells (ESCs) differentiate into round spermatids^29^, showing the potential value of *DAZ* family genes on modulates the development of germ cells. It would be a great breakthrough if we could gain spermatid from the somatic cells derived from azoospermia patients via *DAZ* over-expression. However, in our previous work, after transfecting DAZ over-expressing vectors into fibroblast and Sertoli cells, it was showed that RNA-binding proteins alone were not sufficient to change the features of somatic cells (data not published). It is probably because RNA-binding proteins achieve their function via binding with their target RNAs, while undifferentiated ESCs share a pre-meiotic transcriptional expression profile with germ cells and could differentiate into germ cells spontaneously^30^. The over-expressing of DAZ promoted the translation of these transcripts, shifting the balance to promote the differentiation of these cells into germ cells. While, these target RNAs are not existed in somatic cells, so over-expressing of DAZ genes is not sufficient to induce somatic cells into germ cells.

In conclusion, to initial gene expressions for molding cell via regulating the expression of limited genes, other factors such as transcription factors would be the ideal candidates.

To screen the key transcription factors from thousands of differential expressed genes detected via sequencing, GSEA was carried out to show enriched features of the differential expressed gene set.

NFATs (nuclear factor of activated T-cells protein family) influence the most target genes up-regulated in undifferentiated spermatogonias. A total of 73 out of 476 target genes were annotated to be regulated by NFATs. SP1 influence the most targets genes down-regulated in undifferentiated spermatogonias. A total of 8 out of 109 target genes were annotated to be regulated by SP1. Besides NFATs and SP1, LEF1, TCF3, MAZ, JUN, PAX4, and MLLT7 could also be considered as the potential key transcription factors in undifferentiated spermatogonias.

In primary spermatocytes, SP1 influence the most target genes up-regulated. A total of 21 out of 195 target genes were annotated to be regulated by SP1. TCF3 influence the most targets genes down-regulated in primary spermatocytes. A total of 13 out of 45 target genes were annotated to be regulated by TCF3. Besides SP1 and TCF3, MAZ, LEF1, PAX4, MLLT7, MYOD1, TCF8, and NFAT could also be considered as the potential key transcription factors in spermatocytes.

In spermatid, TCF3 and SP1 play the most critical role as transcription factors. A total of 118 genes out of 1,384 spermtaid up-regulated target genes were annotated with TCF3 target motif features while a total of 154 genes out of 976 spermatid down-regulated target gens were annotated with SP1 target motif features. Besides TCF3 and SP1, LEF1, PAX4, MLLT7, MAZ, JUN, NFAT, and REPIN1 could also be considered as the potential key transcription factors in spermatids.

Transcription factor genes with a RPKM (Reads Per Kilobase per Million mapped reads) value >10 were considered to have an effective expression. According this standard, among the transcription factors mentioned above, MAZ, NFATs, LEF1, SP1, TCF3, JUN and TCF8 were considered to have an effective expression. Among these genes, NFATC4, NFAT5, LEF1, and SP1 have a relatively high expression level in spermatocytes, NFATC1, NFATC2, TCF3, and JUN have a relatively high expression level in undifferentiated spermatogonias, NFATC3 has a relatively high expression level in both spermatocytes and spermatids, TCF8 has a relatively high expression level in both undifferentiated spermatogonias and spermatids (see Tab. S2),

The members of NFAT (Nuclear Factor of Activated T-Cells) transcription factor family play an important role in immune response^31^. Due to their distribution in ovary and testis, NFATs were supposed to be involved in reproduction events though their roles in reproduction were not clear^31^. NFATs could bind cooperatively with transcription factors of the AP-1 family, which consisted with Fos and Jun^32^, thus connected the functions of NFATs with JUN family, which was also considered to be a potential key transcription factor for the gene expression regulation in undifferentiated spermatogonias.

JUN, along with the other two members of JUN family, JUNB and JUND, is a proto-oncogene, also known as AP-1. The abnormal expression of JUN was reported to be related with a variety of tumorigenesis^33^. And it also plays a role in the specification of some cells^34^,^35^. Whether it play a role in the regulation of spermatogenesis was not clear. When phosphorylated by HIPK3, Jun could promote the activity of NR5A1 which cause the increasing of steroidogenic gene expression^36^, while steroid hormones are important for the regulation of spermatogenesis. The expression of *JUN* family genes, including *JUN*, *JUNB*, and *JUND*, in undifferentiated spermatogonias was significantly higher than that in spermatocytes and spermatids, and coincidently, JUN dimerization protein 2 (JDP2) which regulate the transactivation of JUNs^37^ was also up-regulated in undifferentiated spermatognias (Tab. S2). This further indicated that JUN genes are important to undifferentiated spermatogonias.

**Figure 6:**
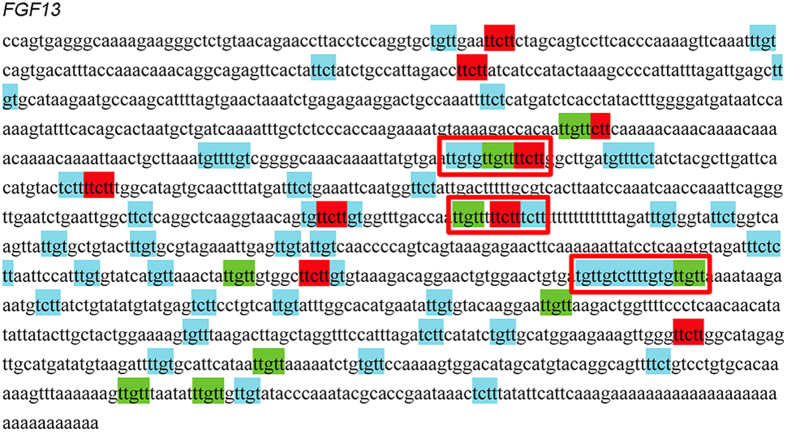
The recognition of potentially DAZ recognized sequence. *FGF13* was used as an example. The simplified minimum unit of *DAZ* target sequence (UUCU, UCUU, UGUU, and UUGU) was used to search for pattern U_2–10_(G/C)U_2–10_ on 3’ UTR sequence of *FGF13*, the tandem units were mostly possible to be the recognition targets of *DAZ* genes which were labeled using red boxes.

As JUN is a ubiquitously expressed transcription factor, promoting the expression of hundred of genes with variety of functions, the post-transcription regulation of JUN downstream genes is essential for the function modulation of specific cells. One widely known post-transcription regulation factor specifically expressed in germ line, as mentioned above, is DAZ family. These RNA-binding proteins recognize specific sequences located at the 3’ UTR of target mRNAs, stabilizing these mRNAs and promoting the translation of them^38^. Up to now, the binding motif for human DAZ genes had not been revealed. Considering that the phenotype of *Dazl* defect mice could be rescued by DAZ^39^, it could be speculated that the binding motif for human *DAZ* genes and mouse *Dazl* were evolutionally conserved. It has been demonstrated that the mouse Dazl target genes shared a common U_2–10_(G/C)U_2–10_ binding motif 38,40. Such pattern was employed to screen the 3’ UTR of the 61 up-regulated target genes potentially regulated by JUN in undifferentiated spermatogonias (Fig. 6). A total of 30 genes were recognized as the potential target genes of DAZ genes. Among these genes, important cytokines, receptors, and transcription factors that involved in spermatogensis, such as BMP2, FGF13, PRDM1 (BLIMP1), TGFBR2, and ZNF503, could be found.

BMP2 belongs to bone morphogenetic protein family. Many members of this family have been proved to play regulatory role during spermatogenesis. It was demonstrated that BMP2 acts as proliferative signals upon spermatogonia during the early postnatal development of mouse testis. BMP2 was reported to increase speramtogonia proliferation^41^.

Blimp1 is the earliest known marker of PGC specification^42^. Blimp1 mutation results in the formation of PGC-like cells at embryonic day 8.5 closely resemble the neighbouring somatic cells^43^. A central role of Blimp 1 is the induction of Tcfap2c^44^. Tcfap2c mutants exhibited a loss of primordial germ cells in early embryos^45^,^46^. Tcfap2c is thought to repress somatic gene expression^46^. So, Blimp1 and Tcfap2c are able to modulate the transcription of necessary genes to regulate PGC specification.

Since the shared target genes of JUN and DAZ genes are so crucial for germ line development, we speculate that JUN and DAZ genes had some synergetic effect that determined the fate of germ cells. Such hypothesis remained to be proved.

SP1 (Specificity Protein 1) was considered as a general transcription factor for it is required for transcription of a series house-keeping genes^47^. SP1 is involved in many cellular processes, including cell differentiation^48^,^49^, cell growth^47^, apoptosis^50^, immune responses^51^, response to DNA damage^52^, and chromatin remolding^53^. During spermatogenesis, great changes occurred on chromosome, like successive chromosome replication, chromosome synapsis, chromosome segregation, and chromosome condensation. SP1 may play an important role in these processes, especially in the meiosis chromatin remolding for its relative high expression level in spermatocytes.

TCF3 (Transcriptin Factor 3) was reported to be involved in the initiation of neuronal differentiation via repressing Wnt signaling Pathway^54^. It was proved that the Wnt signaling pathway is critical for postnaltal testis functions^55^,^56^. Active canonical Wnt signaling could be identified through nuclear localization of β-catenin^57^,^58^ which could be detected in stage VI pachytene spermatocytes and increased with the process of spermatogeneiss. The most prominent nuclear staining was detected in stage I-VIII round spermatids. Soon after that, β-catenin signal was eliminated from the nuclear with the elongating of the spermatids^59^. Aberrant up-regulation of Wnt signaling results in germ cell loss, especially in the postmitotic germ cells^59^. Defect of the Wnt antagonist naked cuticle 1 (Nkd1) have reduced fertility in mice, and the non-functional Nkd1 transcript was shown to be present in spermatids at increasing levels from round to elongating spermatids^60^. Coincidently, Nkd1 is one of the target genes regulated by Tcf3 (see http://www.broadinstitute.org/gsea/msigdb/download_file.jsp?filePath=/resources/msigdb/5.0/c3.tft.v5.0.symbols.gmt). These findings suggested that shutting down Wnt signaling pathway at proper timing is essential for spermatogenesis, especially critical to the elongating of spermatids. TCF3 may play a role in spermiogenesis via regulating the activity of Wnt signaling pathway just like that in neuronal differentiation.

In current work, the potential key transcription factors for spermatogenesis were screened via GSEA. These transcription factors, along with some other transcription factors with specific expression patterns, for example, the specifically expression of *HOX*s in spermatocytes, were considered to be important to the three crucial stages of male gametes generation. The defect or misarranged expression of these transcription factors could be the putative cause of spermatogenesis dysfunction which leads to male infertility. Meanwhile, these genes would be the very candidate that could be used in the modulation of cell features to produce germ cells for those who suffering from germ cell loss. However, it should be noticed that the roles of these genes during spermatogensis still need to be confirmed via further investigation before them be applied as our molecular tools.

## Methods

### Ethics statement

This study was approved by the Institutional Ethical Review Committee of Ren Ji Hospital, Shanghai Jiao Tong University, School of Medicine (license number of ethics statement: 2012-01). An informed consent of testis tissues used for research only was obtained from each included patient. All experiments were performed in accordance with relevant guidelines and regulations of the Institutional Ethical Review Committee of Ren Ji Hospital.

### Isolation of germ cells

Testis tissues were obtained from patients with OA in which case the spermatogenesis was thought to be normal via surgery. A total of 27 patients were included in this work (tab. S3).

Two-step enzymatic digestion was applied to isolate germ cells from testis. The testis tissues were cut into pieces and incubated with 10 mL Enzyme I (2 mg/mL type IV Collagenase (17104-01, Gibco) and 10 μg/mL DNase I (D4527, Sigma) in DMEM (SH30243.01B, HyClone)) in oscillating water bath at 34 °C, 100 rpm for 10–15 min. The seminiferous tubules were collected via removing the supernatant after sedimentation for 10 min at room temperature and incubated with 10 mL Enzyme II (mg/mL type IV collagenase (17104-01, Gibco), 2 mg/mL Hyaluronidase (H3506, Sigma), 2 mg/mL Pancreatin (T8003, Sigma) and 10 μg/mL DNase I (D4527, Sigma) in DMEM (SH30243.01B, HyClone)) in oscillating water bath at 34 °C, 100 rpm for 10–15 min. The suspension was centrifuged at 300 g for 5 min and the supernatant was then removed. The precipitant was re-suspended using DF12-FBS (10% FBS in DF12) followed by being filtered using a 40 μM cell strainer and planted in a 10 cm dish which was coated with gelatin.

### Ploidy sorting via FACS

A Flow Cytometer (FACS Aria II Flow Cytometer, BD Biosciences) was employed to sort germ cells into haploid, diploid and tetraploid. The testicular cells were cultured at 34 °C with 5% CO_2_ in an incubator for 48 hours to ensure the somatic cells adhesion to the dish thoroughly. The unbinding cells were collected by pipetting gently. After being centrifuged at 300 g for 5 min and removing the supernatant, the collected cells were suspended in incubating buffer (DF12 with 0.5% BSA and 2 mM EDTA) followed by adding Hochest 33342 (14533, Sigma) to a concentration of 5 μg/mL. After incubating in a cell incubator at 34 °C for 40 min, the suspension was filtered with a 40 μM cell strainer and loaded to the Flow Cytometer for sorting. The haploid, diploid and tetraploid cells were collected respectively. After being centrifuged and removing the supernatant, the diploid cells were suspended with DF12-FBS, and replanted into the dish. The haploid and tetraploid cells, which were putative spermatids and primary spermatocytes, were washed by PBS once and the cell pellets were sheared using a lysis buffer offered by Qiagen kit while a portion of these cells were made cytospin after fixed by 4% PFA for identification.

### Sorting of CD90+ cells via MACS

MACS was used to isolated CD90+ cells which were believed to be enriched undifferentiated spermatogonias. FACS sorted diploid cells were collected via centrifuged at 300 g for 5 min and suspended in 50 μL incubating buffer. Appropriate amount of anti-CD90 antibody labeled MACS micro beat (130-096-253, Miltenyi) was added and the cell suspension were incubated at 4 °C for 20 min. The MACS Separation Column was put into the magnetic field and balanced via loading with 500 μL incubating buffer. Then the incubated cell suspension was mixed with 500 μL incubating buffer and loaded onto the column. The column was then washed by incubating buffer for at least three times. The effluent was checked using microscope to make sure that all negative cells had been removed from the column. Then the magnetic field was removed and the positive cells were collected. The column was washed by washing buffer (DF12 with 2% FBS and 2 mM EDTA) to make sure as many positive cells were collected as possible. The collected cells were sheared using a lysis buffer offered by Qiagen kit while a portion of these cells were made cytospin after fixed by 4% PFA for identification.

### Immunofluorescence for identification of cell type

Immunofluorescence was performed to identify the sorted cells. PFA fixed Cytospin was treated with 4% Triton X-100 for 20 min followed by blocked in 5% BSA for 1 hour at room temperature. Primary antibody was diluted in BSA blocking buffer at a dilution ration of 1:200. The cytospin was incubated with the diluted primary antibody at 4 °C over night followed by washing with PBS for three times, 20 min each. Secondary antibody was diluted in BSA blocking buffer at a dilution ration of 1:200 with 0.1 μg/mL DAPI. The cytospin was then incubated with the diluted secondary antibody at 37 °C for 1 hour followed by washing with PBS for four times, 20 min each. A Dako fluorescence mounting medium was used to mount the slide and then a fluorescence microscope (Leica) was used to observe the cytospin.

### Meiosis spreads

Meiosis spread assay was performed to determine the meiosis progression in the FACS sorted tetraploid cells. Briefly, cells were lysed by a hypotonic solution and spread evenly over slides layered with 1% PFA and 0.15% Triton X-100. Slides were dried for 24 hours at room temperature in a humid chamber. The cells were treated with 0.04% photoflo for 5 min and blocked with 4% goat serum. Staining was performed in cells incubated with primary antibodies anti-SCP3 (ab15093, Abcam) overnight at 37 °C in a humid chamber. Donkey Anti-Rabbit IgG (H + L)(Alexa Fluor 555) (A31572, Molecular Probes) were applied at 1:1000 dilution and incubated for 90 min at 37 °C. Cells were washed three times with PBS and counterstained with DAPI, and images were captured with a fluorescence microscope.

### RNA extraction and sequencing

A Qiagen RNeasy Micro Kit (74004, Qiagen) was used to extract RNA from the sorted cells. The extraction was performed according to Quick-Start Protocol of RNeasy Micro Kit. The integrity and quality of the extracted RNAs were checked by Agelient 2100 bioanalyzer and the qualified RNA samples were used to be sequenced. For spermatids and primary spermatocytes, every 5 samples were pooled according to the RNA quantity. A total of 3 pools were prepared for each type of cells, so we had 3 biological replicates. Dynabeads mRNA DIRECTTM kit (610.12, Life technologies) was used to enrich RNAs with polyA tail. mRNA-seq library were prepared using TruSeq RNA kit (RS-122-2001, illumina). Sequencing was performed on Illumina Hiseq 2500 next generation sequencing platform.

While the amount of undifferentiated spermatogonias were rare, SMARTer Ultra Low Input RNAkit for sequencing-v3 (634848, Clontech Laboratories) was used to amplification the cDNA derived from these cells for sequencing purpose before sequencing was performed.

### Raw data production and preprocessing

TopHat (v2.0.8b, http://tophat.cbcb.umd.edu/) were used to map the RNA-seq reads to human genome build hg19 (UCSC). The reads with low quality were removed from the raw sequencing reads. Read mapping were performed using Tophat (R software), reads count were obtained using HTSeq (http://www-huber.embl.de/users/anders/HTSeq/doc/overview.html). Differential expressed genes were analysis using DESeq R software pack. Benjamini-Hochberg multiple testing correction was employed to reveal the differential expressed genes.

### Q-PCR verification of sequencing results

Q-PCR was employed to verify the sequencing results. A total of 41 differential expressed genes from cluster 1 to cluster 4 were chosen for verification purpose (Tab. S4). ABI Power SYBR green PCR Master Mix (4367659, applied biosystems) was used to performed Q-PCR on ABI StepOne Plus Realtime-PCR System. ACTB was used as the internal reference.

### Immunohistochemistry

Paraffin-embedded sections of testis tissues derived from OA patients were prepared. The sections were baked at 80 °C for 15 min and treated with fresh Xylene 3 times, 10 min each, to remove the paraffin. Then, the sections were immersed sequentially in 100%, 95%, 85% and 75% ethanol for 20 min each to be rehydrated. The rehydrated sections were washed with water, and 0.01 M citrate buffer (9 ml 0.1 M citric acid, 41 ml sodium citrate, add ddH2O to 500 ml, pH 6.0) was used for antigen retrieval in a pressure-cooker. The treated sections were then cooled to room temperature naturally followed by treated with 0.4% Triton X-100 for 20 min. Removing the Triton solution, 0.3% H_2_O_2_ was used to incubate the sections for 15 min to inactive the endogenous catalase. The sections were then blocked with 5% BSA/PBS at room temperature for 1 h followed by incubated with primary antibody solution at 4 °C overnight. A total of 6 antibody were used to detect the corresponding proteins, included Rabbit anti-RFX1 antibody (NBP1-52654, Novus biologicals) with a dilution of 1:1000, mouse anti-Hsp60 antibody (ab13532, abcam) with a dilution of 1:100, Rabbit anti-GRIK5 antibody (ab67408, abcam) with a dilution of 1:150, rabbit anti-TRIM66 antibody (PAB21896, Abnova) with a dilution of 1:100, rabbit anti-NFKBIZ antibody (PAB20499, Abnova) with a dilution of 1:150, and rabbit anti PHF16 antibody (PA5-28533, Thermo Scientific) with a dilution of 1:500. All antibodies were diluted in 5% BSA/PBS. Normal rabbit IgG was used as negative control.

After incubation with primary antibody, the sections were washed with PBS 3 times for 15 min each, and then incubated with HRP-labeled anti-rabbit & mouse antibody provided by GTVsion III Detection System Kit (GK500705, Gene Tech) at room temperature for 30 min followed by washing with PBS 3 times for 15 min each. DAB staining solution provided by the kit was used to stain the treated sections according to the instructions. After staining, the sections were washed with water and Gill’s hematoxylin dye was used (2 g hematoxylin, 250 ml Ethanol, 17 g aluminum potassium sulfate, 750 ml ddH2O, 0.2 g sodium iodide, 20 ml glacial acetic acid) to stain the nucleus.

### Expression pattern cluster

The differential expressed genes were clustered according to their expression pattern.

### GO annotation and pathway analysis

Genes with different expression patterns were made GO annotation with Blast2GO respectively. Pathway analysis was performed by uploading these genes to KEGG (http://www.genome.jp/kegg/).

### Gene set enrichment analysis

GSEA was performed to gain the stage specific transcription factor targets enrichment features of spermatogenesis.

## Additional Information

**How to cite this article**: Zhu, Z. *et al.* Dynamics of the Transcriptome during Human Spermatogenesis: Predicting the Potential Key Genes Regulating Male Gametes Generation. *Sci. Rep.* **6**, 19069; doi: 10.1038/srep19069 (2016).

## Supplementary Material

Supplementary Information

## Figures and Tables

**Table 1:** The potential key transcription factor involved in spermatogenesis.

Targets Expression Pattern	Transcription Factor	Target Count	Targets Expression Pattern	Transcription Factor	Target Count
SPG up-regulated	NFAT	73	SPG down-regulated	SP1	8
	LEF1	69		LEF1	6
	SP1	69		PAX4	5
	TCF3	65		unknown^*^	5
	MAZ	63		unknown^*^	5
	JUN	61		NRF1	5
	unknown^*^	58		TCF3	4
	PAX4	57		NFAT	4
	MLLT7	56		GATA1	4
	MYC	42		HNF4A	4
SPC up-regulated	SP1	21	SPC down-regulated	TCF3	13
	MAZ	19		MAZ	8
	LEF1	18		SP1	7
	TCF3	15		LEF1	6
	PAX4	15		JUN	6
	MLLT7	13		MYOD1	6
	TCF8	13		unknown^*^	6
	unknown^*^	13		NFAR	5
	unknown^*^	12		MLLT7	5
	NFAT	10		REPIN1	5
				TFAP4	5
SPT up-regulated	TCF3	118	SPT down-regulated	SP1	154
	LEF1	106		LEF1	103
	unknown^*^	90		TCF3	92
	PAX4	84		MAZ	84
	MLLT7	84		unknown^*^	83
	MAZ	81		ELK1	82
	SP1	77		NFAT	76
	JUN	65		MLLT7	65
	NFAT	61		MYC	64
	REPIN1	66		REPIN1	51

**Table 2:** Differential expressed non-coding RNAs.

Cluster	Count	Genes
6	10	ASZ1_lncRNA, DBF4B_lncRNA, ELOVL2-AS1, LINC00552, LINC00661, LINC00668, NBPF3_lncRNA, PES1_lncRNA, PILRB_lncRNA, SNAI3-AS1
7	7	FGD5-AS1, ILF3-AS1, MAP3K13_lncRNA, NGRN_lncRNA, SETD5-AS1, TP73-AS1, TRAF3IP2-AS1
8	77	ARHGAP26-AS1, BRWD1-AS1, C20orf173_lncRNA, CAST_lncRNA, CDKN2B-AS1, CELF2-AS2, CSNK1G2-AS1, DACT3-AS1, DIAPH3-AS1, DIAPH3-AS2, DNAJB8-AS1, FAM170B-AS1, FAM181A-AS1, FAM222A-AS1, GRID1-AS1, IGSF11-AS1, ISM1-AS1, KCNQ5-AS1, KDM5B-AS1, KIAA1984-AS1, LINC00085, LINC00112, LINC00158, LINC00174, LINC00226, LINC00254, LINC00265, LINC00271, LINC00277, LINC00282, LINC00293, LINC00301, LINC00347, LINC00442, LINC00467, LINC00494, LINC00521, LINC00550, LINC00559, LINC00592, LINC00598, LINC00606, LINC00608, LINC00616, LINC00633, LINC00635, LINC00642, LINC00658, LINC00691, LINC00700, LINC00705, LINC00710, LINC00838, LINC00841, LINC00851, LINC00854, LNX1-AS1, LSAMP-AS3, MACROD2-AS1, MAP3K14-AS1, MKNK1-AS1, MRVI1-AS1, NAALADL2-AS3, NAV2-AS5, NPPA-AS1, NTRK3-AS1, OSBPL10-AS1, SATB2-AS1, SHANK2-AS1, SPTY2D1-AS1, SRD5A3-AS1, STXBP5-AS1, TEX26-AS1, UBOX5-AS1, VPS13A-AS1, WDR52-AS1, ZNF32-AS3
9	4	LINC00202-1, LINC00202-2, LINC00264, LINC00654
10	2	LINC00152, MAGI2-AS3
11	20	DLG1-AS1, GNG12-AS1, HAS2-AS1, KIAA1967_lncRNA, KIRREL3-AS2, LINC00229, LINC00238, LINC00326, LINC00340, LINC00535, LINC00634, LINC00643, PITRM1-AS1, PSMD6-AS2, PVRL3-AS1, STARD13-AS, TSLP_lncRNA, TTN-AS1, ZMIZ1-AS1, ZNF295-AS1
12	14	FAM193B_lncRNA, GABPB1-AS1, GOLGA8B_lncRNA, KIF9-AS1, LINC00641, MCM3AP-AS1, MLK7-AS1, PSMG3-AS1, PSPC1_lncRNA, RSBN1L-AS1, SRSF10_lncRNA, TTC28-AS1, UNK_lncRNA, WDFY3-AS2
14	2	LINC00624, MFI2-AS1
15	19	BSN-AS2, BVES-AS1, HOXD-AS1, LEF1-AS1, LINC00029, LINC00221, LINC00343, LINC00353, LINC00662, PAXBP1-AS1, PCYT2_lncRNA, PPP1R1C_lncRNA, PROSER2-AS1, PVRL3-AS1, RNF157-AS1, SRRM2-AS1, TIPARP-AS1, TPT1-AS1, ZNRD1-AS1
16	2	LINC00294, SARS_lncRNA
