# Supplementary material for: Dynamics of the Transcriptome during Human Spermatogenesis: Predicting the Potential Key Genes Regulating Male Gametes Generation
Source: Sci Rep. 2016 Jan 12;6:19069. doi: 10.1038/srep19069 (PMC4750114; doi:10.1038/srep19069)
Supplement: Supplementary Information [file srep19069-s1.doc]

**Dynamics of the Transcriptome during Human Spermatogenesis: Predicting the Potential Key Genes Regulating Male Gametes Generation**

*Zijue Zhu^1,2^, Chong Li^3^, Shi Yang^1^, Ruhui Tian^1,2^, Junlong Wang^1^, Qingqing Yuan^4^, Hui Dong^3^, Zuping He^4^, Shengyue Wang^3^, and Zheng Li^1,2,5*^*

*1 Department of Urology, Ren Ji Hospital, School of Medicine, Shanghai Jiao Tong University, 1630 Dongfang Road, Shanghai 200127, China*

*2 Department of Andrology, Urologic Medical Center, Shanghai General Hospital,, Shanghai Jiao Tong University, 100 Haining Road, Shanghai 200080, China*

*3 Shanghai-MOST Key Laboratory of Health and Disease Genomics, Chinese National Human Genome Center at Shanghai, 250 Bibo Road, Shanghai 201203, China*

*4 Clinical Stem Cell Research Center, Ren Ji Hospital, School of Medicine, Shanghai Jiao Tong University, 1630 Dongfang Road, Shanghai 200127, China*

*5 Shanghai Key Laboratory of Reproductive Medicine, Shanghai, 200025, China*

*Correspondence: Zheng Li, M.D. & Professor, Department of Urology, Ren Ji Hospital, School of Medicine, Shanghai Jiao Tong University, 1630 Dongfang Road, Shanghai 200127, China, E-mail: li[zhengboshi@163.com](mailto:zhengboshi@163.com); Tel：86-135-6478-3816

Supplementary Table 1 the pathways that stage specific up-regulated genes mainly involved in

| Expression Pattern | Pathway | Sequence in Pathway | Gene Name |
| --- | --- | --- | --- |
| SPG up-regulated | Purine metabolism | 26 | *ATP1B1, TOR3A, ARL8A, RAB38, GNB5, RAB31, ARL4C, GNL3, FGF2, RHOBTB3, TAP2, TAP1, TAPBP, RAB23, AGAP3, TOR1A, GBP3, GBP1, RRAD, MRAS, DCTPP1, ADK, AK3, PRPS2, PNP, RUNX1* |
|  | Cytokine-cytokine receptor interaction | 23 | *LIF,ACVR1,CCR7,CCL2,CXCL3,IL13RA1,CXCL2,VEGFA,PDGFRA,NGFR,TGFBR2,LTBR,BMP2,TNFRSF1B,IL7R,IL6,IL24,IL8,TNFRSF1A,KITLG,TNFRSF12A,TNFRSF10D,INHBA* |
|  | Pathways in cancer | 22 | *MMP9,TPM3,COL4A2,FGF13,FGF2,MMP1,CDKN1A,VEGFA,PDGFRA,GSTP1,BID,TGFBR2,FGFR1,BMP2,PTGS2,MYC,IL6,IL8,NFKBIA,KITLG,RUNX1,ITGAV* |
|  | MAPK signaling pathway | 14 | *PLA2G4A,DUSP2,GNG12,DUSP6,FGF13,FGF2,MYC,MAP3K8,TNFRSF1A,MRAS,PDGFRA,NGF,TGFBR2,FGFR1* |
|  | Lysosome | 12 | *SCARB2,CD68,CTSS,CTSO,CD63,CTSB,HEXB,LAPTM5,LAPTM4B,HGSNAT,ARSB,NEU1* |
|  | Focal adhesion | 12 | *ITGB8,COL4A2,VAV2,CAV1,PARVA,ITGAV,VEGFA,PDGFRA,SRC,MYL12A,CAV2,THBS1* |
|  | Chemokine signaling pathway | 11 | *GNG12,GNB5,CCR7,CCL2,CXCL3,VAV2,NFKBIA,IL8,CXCL2,LYN,FOXO3* |
|  | Regulation of actin cytoskeleton | 11 | *GNG12,ITGB8,FGF13,FGF2,VAV2,MRAS,ITGAV,PDGFRA,MSN,MYL12A,FGFR1* |
|  | TGF-beta signaling pathway | 9 | *DCN,BMP2,ACVR1,MYC,ID2,FST,TGFBR2,INHBA,THBS1* |
|  | Phagosome | 9 | *CTSS,COLEC12,SEC61A1,TAP1,STX7,TAP2,C1R,ITGAV,THBS1* |
|  | Tuberculosis | 9 | *LTBR,PLK3,CTSS,IL6,TNFRSF1A,SPHK1,BID,SRC,CD74* |
|  | Jak-STAT signaling pathway | 8 | *LIF,IL7R,IL6,MYC,IL24,IL13RA1,SPRY4,SPRY2* |
| SPC up-regulated | Purine metabolism | 15 | *MSH4, MYO1A, DNM1P46, SRCAP, RNF112, TOP2A, DDX17, DMC1, KIF20A, PMS2P5, ATP8B5P, PPP2R4, SETX, POLR2A, POLE* |
|  | Progesterone-mediated oocyte maturation | 3 | *SPDYA,BUB1,CCNB3* |
|  | Oocyte meiosis | 3 | *SMC1B,SPDYA,BUB1* |
|  | Cell cycle | 3 | *SMC1B,BUB1,CCNB3* |
| SPT up-regulated | Purine metabolism | 39 | *ALLC, LINC00282, ATP10A, ATP6V1E2, CHD5, ATP1A2, ATP1A4, ATP7B, DNAH9, KIF2B, DHX57, ABCB11, ABCA12, LOC100131047, DNAH11, PDE4A, PDE11A, PDE1A, PDE6A, PDE1C, AK8, ADCY8, NT5C1B, EHD1, GNG2, TUBA4A, KIF17, DNALI1, MYO7A, MYH7, STARD9, DYNLRB2, MYH1, RAB27B, KIF5C, MYH7B, TUBA8, MYO18B, KIF15* |
|  | Neuroactive ligand-receptor interaction | 17 | *VIPR1,PTH1R,P2RX3,GRID1,CHRM4,OPRM1,GRM3,GLRA3,GRIK1,GRM5,GRIN2B,GRM7,GRID2,OPRL1,CHRNB4,GPR156,GABRG3* |
|  | Calcium signaling pathway | 12 | *PDE1A,CACNA1C,ADCY8,P2RX3,PLCD4,PLCG2,CACNA1A,PLCE1,PDE1C,CAMK4,GRM5,NOS3* |
|  | Glutamatergic synapse | 11 | *GNG2,CACNA1C,ADCY8,GRM7,SHANK2,CACNA1A,GRM3,GRIK1,GRM5,PLA2G4E,GRIN2B* |
|  | MAPK signaling pathway | 11 | *MAPKAPK2,HSPA1L,MKNK1,FGF14,CACNA1C,CACNA1A,MAP3K14,IL1A,MAPK10,MAPK8IP1,PLA2G4E* |
|  | Pathways in cancer | 11 | *CDKN2B,FGF14,DCC,PLCG2,FZD9,WNT7A,TRAF4,MAPK10,CDKN2A,CTNNA2,LAMB4* |
|  | Axon guidance | 10 | *EPHA6,EFNB3,ROBO2,DCC,SLIT3,EPHA1,SLIT1,NTN1,SLIT2,UNC5D* |
|  | Tight junction | 10 | *EPB41L3,MYH7,PARD6A,IGSF5,PPP2R2C,MAGI1,SYMPK,MYH1,MYH7B,CTNNA2* |
|  | Regulation of actin cytoskeleton | 10 | *FGF14,FGD3,CHRM4,GIT1,DIAPH3,PFN3,TIAM2,ARHGAP35,IQGAP2,PIP5K1B* |
|  | Pancreatic secretion | 9 | *SLC26A3,CA2,SLC4A4,ADCY8,KCNMA1,ATP1A4,RAB27B,ATP1A2,PLA2G4E* |
|  | Insulin signaling pathway | 9 | *ACACB,MKNK1,SLC2A4,INPP5D,LIPE,HK1,MAPK10,G6PC2,HK3* |
|  | Bile secretion | 8 | *CA2,SLC4A4,ADCY8,ATP1A4,ABCB11,SLC51B,SLC27A5,ATP1A2* |

Supplementary Table 2 RPKM Value of the genes discussed in the discussion section

|  | RPKM Value | | | | | | | | |
| --- | --- | --- | --- | --- | --- | --- | --- | --- | --- |
| Gene | SPG-Pool1 | SPG-Pool2 | SPG-Pool3 | SPC-Pool1 | SPC-Pool2 | SPC-Pool3 | SPT-Pool1 | SPT-Pool2 | SPT-Pool3 |
| *ACSL4* | 22.61037 | 45.75796 | 35.53706 | 2.515631 | 1.290839 | 2.317335 | 0.185499 | 0.233752 | 0.15638 |
| *BMP2* | 12.68174 | 12.38319 | 15.55856 | 0.292713 | 0.187879 | 0.350096 | 0.148358 | 0.126686 | 0.123598 |
| *CAMKK1* | 4.278999 | 6.238564 | 3.529378 | 0.878576 | 0.415249 | 0.337605 | 0.079948 | 0.072536 | 0.04355 |
| *CCR7* | 30.69551 | 20.00569 | 13.23801 | 0.250182 | 1.022332 | 0.239185 | 0.541239 | 0.616683 | 0.241934 |
| *CDKN1A* | 91.98789 | 82.90897 | 141.6352 | 2.108543 | 1.066159 | 0.883829 | 0.756919 | 0.343373 | 0.429269 |
| *CTHRC1* | 22.10542 | 35.8768 | 13.74133 | 0.658037 | 0.500482 | 0.713475 | 0.146126 | 0.016572 | 0.110869 |
| *ELK3* | 42.012 | 67.6431 | 69.7088 | 2.698965 | 1.563278 | 3.130122 | 0.523983 | 0.637739 | 0.840366 |
| *FGF13* | 3.86045 | 3.728458 | 8.484669 | 0.316569 | 0.093514 | 0.193362 | 0.071671 | 0.043351 | 0.032224 |
| *FOXF2* | 3.426644 | 4.271835 | 4.688892 | 0.499678 | 0.405912 | 0.172508 | 0.037709 | 0.011404 | 0 |
| *HS6ST2* | 3.391687 | 8.077553 | 10.19053 | 0.112163 | 0.058903 | 0.076254 | 0.060192 | 0 | 0.012179 |
| *IL6* | 110.5668 | 186.4667 | 227.2133 | 4.297574 | 5.498322 | 1.446152 | 1.671684 | 3.286196 | 0.939516 |
| *IL7R* | 8.288875 | 25.36576 | 31.63465 | 0.200445 | 0.187137 | 0.157723 | 0.131474 | 0.032532 | 0.060456 |
| *ITGB8* | 5.355196 | 20.11483 | 23.23805 | 0.392974 | 0.110337 | 0.234761 | 0.159734 | 0.198917 | 0.3517 |
| *JDP2* | 2.296305 | 6.319935 | 2.977058 | 0.111862 | 0.012588 | 0.00679 | 0.250844 | 0.192575 | 0.091094 |
| *JUN* | 44.50033 | 224.7559 | 83.76441 | 16.86425 | 12.45876 | 5.205106 | 7.528084 | 9.134417 | 6.762024 |
| *JUNB* | 85.33098 | 457.5852 | 173.748 | 7.710043 | 5.332774 | 3.579693 | 2.28578 | 2.018934 | 1.087272 |
| *JUND* | 82.09555 | 202.5716 | 124.8974 | 74.94705 | 38.23666 | 26.24313 | 78.54471 | 60.89966 | 40.46529 |
| *LEF1* | 5.347525 | 2.064916 | 4.702454 | 7.586098 | 20.71003 | 26.66594 | 1.538316 | 2.181163 | 2.095156 |
| *LMO4* | 29.61008 | 36.60489 | 20.574 | 1.730865 | 0.726513 | 1.594388 | 0.325445 | 0.355251 | 0.313798 |
| *MAFF* | 48.0254 | 54.34165 | 102.3197 | 3.941405 | 2.945274 | 2.982404 | 1.749358 | 2.37584 | 2.264344 |
| *MAZ* | 1.917962 | 14.54169 | 1.042916 | 20.0411 | 26.10166 | 15.4184 | 0.566901 | 1.325093 | 0.75818 |
| *MLLT7* | 0.749246 | 1.205552 | 1.895109 | 0.663005 | 0.45799 | 0.485418 | 0.024633 | 0.029799 | 0.008306 |
| *MMP9* | 26.75188 | 69.57193 | 59.87179 | 2.163603 | 2.6256 | 1.292035 | 1.294474 | 1.19582 | 0.976195 |
| *MYOD1* | 0.156259 | 0.468855 | 0.056971 | 0.113455 | 0.104267 | 0.016069 | 0.136993 | 0.193344 | 0.030797 |
| *NFAT5* | 5.55013 | 8.369257 | 17.50187 | 54.45692 | 37.00249 | 54.08765 | 1.10587 | 1.523263 | 2.294419 |
| *NFATC1* | 13.68161 | 6.416567 | 15.20194 | 3.988088 | 4.542433 | 4.242702 | 6.273217 | 5.372323 | 6.105616 |
| *NFATC2* | 3.297662 | 1.766556 | 14.66953 | 0.490545 | 0.289813 | 0.279156 | 0.754501 | 1.071613 | 1.341084 |
| *NFATC3* | 23.35477 | 15.25668 | 15.5687 | 62.39609 | 44.33595 | 45.37964 | 48.03678 | 56.27006 | 61.91432 |
| *NFATC4* | 2.123357 | 4.848624 | 1.889857 | 41.4786 | 44.26479 | 33.29357 | 5.342853 | 10.54783 | 9.706841 |
| *NKD1* | 2.860024 | 0.838721 | 1.172592 | 1.966868 | 1.428994 | 1.594894 | 5.181956 | 4.509824 | 3.764961 |
| *NLN* | 4.300988 | 7.90928 | 6.612966 | 0.989679 | 0.543546 | 0.639999 | 0.076176 | 0.040316 | 0.03532 |
| *PAX4* | 0.920398 | 0.308931 | 0.170629 | 1.580068 | 1.057298 | 0.693041 | 2.940463 | 2.655451 | 1.023838 |
| *PERP* | 7.252246 | 2.972097 | 7.143079 | 0.420721 | 0.306402 | 0.290076 | 0.134191 | 0.075369 | 0.058179 |
| *PRDM1* | 3.177417 | 4.841872 | 3.608804 | 0.215097 | 0.254158 | 0.193546 | 0.300464 | 0.429705 | 0.180315 |
| *REPIN1* | 11.24734 | 26.82688 | 10.91015 | 3.964277 | 7.610276 | 5.715881 | 2.272368 | 4.636969 | 4.514609 |
| *RHBDF1* | 8.991777 | 14.84836 | 16.19677 | 2.522432 | 1.546441 | 1.648925 | 0.339949 | 0.191729 | 0.130125 |
| *RUNX1* | 11.79433 | 21.61176 | 25.04007 | 1.377804 | 0.937031 | 0.821797 | 0.564885 | 0.362511 | 0.324057 |
| *SLC9A7* | 1.493735 | 3.35303 | 3.230023 | 0.180381 | 0.117963 | 0.159075 | 0.065752 | 0.029828 | 0.024944 |
| *SP1* | 11.7595 | 9.885012 | 14.15426 | 31.83859 | 24.39531 | 27.78077 | 15.81616 | 16.43967 | 20.7868 |
| *SQSTM1* | 270.586 | 333.3115 | 453.6555 | 19.03015 | 19.15962 | 13.7798 | 5.207921 | 5.487703 | 3.859674 |
| *STC1* | 20.70446 | 73.03431 | 147.0134 | 0.413991 | 0.437131 | 0.224563 | 0.198534 | 0.135096 | 0.064557 |
| *STC2* | 4.002386 | 7.554158 | 2.971965 | 0.089481 | 0.135939 | 0.086906 | 0.087466 | 0.093361 | 0.083278 |
| *TCF3* | 30.77949 | 31.84523 | 37.72011 | 11.95026 | 9.724111 | 8.300601 | 2.667897 | 3.662833 | 1.884494 |
| *TCF8* | 16.27918 | 15.2722 | 34.06552 | 9.717393 | 5.764174 | 6.66363 | 20.9154 | 15.47076 | 14.99955 |
| *TCFAP2C* | 1.718284 | 0.295551 | 1.209051 | 0.190715 | 0.056337 | 0.141813 | 0.105546 | 0.008706 | 0.02912 |
| *TGFBR2* | 20.35503 | 54.42766 | 83.55678 | 0.522813 | 0.457595 | 0.481319 | 0.379937 | 0.413657 | 0.254267 |
| *THBS1* | 29.81793 | 55.5142 | 33.72178 | 2.996222 | 3.112883 | 3.373263 | 2.108035 | 1.925527 | 2.065312 |
| *TLL1* | 2.814253 | 6.351279 | 3.739655 | 0.043911 | 0.038049 | 0.041048 | 0.070695 | 0.089798 | 0.06079 |
| *TPBG* | 22.59936 | 39.10274 | 52.26814 | 1.600928 | 1.075416 | 1.585852 | 3.012268 | 2.768874 | 3.287251 |
| *XYLT1* | 5.494678 | 6.085807 | 7.934887 | 0.552338 | 0.253564 | 0.317068 | 0.309173 | 0.301883 | 0.148938 |
| *ZNF503* | 4.563911 | 8.189799 | 6.091617 | 0.633399 | 0.262159 | 0.127726 | 0.129629 | 0.086248 | 0.069939 |
| *SYCP1* | 15.88461 | 23.12192 | 9.728763 | 338.6902 | 239.703 | 352.6935 | 28.13581 | 30.3656 | 58.33654 |
| *SYCP2* | 21.00505 | 11.42395 | 13.95636 | 152.0541 | 129.031 | 203.8202 | 26.60201 | 34.39454 | 68.08293 |
| *ODF1* | 44.98347 | 19.90521 | 6.383123 | 293.675 | 118.2123 | 102.6833 | 1149.432 | 786.005 | 658.0874 |
| *PRM3* | 35.71939 | 23.38389 | 8.65733 | 20.33518 | 17.58569 | 9.579776 | 109.4259 | 102.2678 | 46.97943 |
| *MAGEA4* | 114.4692 | 20.57327 | 95.90697 | 30.46411 | 13.361 | 15.67584 | 0.94211 | 0.829253 | 1.066867 |
| *UCHL1* | 69.46289 | 122.3673 | 83.42189 | 18.19907 | 10.00406 | 9.140093 | 1.589719 | 1.46453 | 0.841218 |
| *TCAM1P* | 2.297104 | 2.756987 | 1.602633 | 19.34506 | 44.78344 | 43.01105 | 1.408889 | 2.789646 | 2.146299 |
| *TEX101* | 12.92824 | 30.1403 | 11.87257 | 305.1885 | 273.0463 | 230.2662 | 8.89984 | 7.773566 | 8.411663 |
| *ADAM2* | 8.924931 | 4.66857 | 2.609222 | 19.78933 | 112.0327 | 167.1294 | 17.46993 | 34.28733 | 51.84049 |
| *ACR* | 6.179638 | 2.060225 | 0.776049 | 76.40094 | 129.8115 | 103.6709 | 182.4768 | 223.2163 | 204.6954 |
| *ACRBP* | 325.5132 | 111.4487 | 29.16826 | 273.1083 | 399.5908 | 327.3105 | 834.23 | 899.0272 | 745.0217 |
| *CATSPER2* | 25.2423 | 6.783756 | 4.513692 | 36.32663 | 58.76436 | 54.27716 | 27.43838 | 36.56574 | 29.81171 |
| *PGK2* | 373.3027 | 79.39766 | 32.93667 | 198.736 | 171.6881 | 156.9714 | 755.5497 | 622.8263 | 624.5984 |
| *LDHC* | 519.522 | 102.7666 | 57.69479 | 304.5826 | 657.9612 | 715.6274 | 512.4171 | 598.3796 | 749.3881 |
| *TCP10* | 2.315364 | 0.864712 | 0.350238 | 5.338429 | 18.97643 | 14.20278 | 3.735864 | 5.916967 | 3.014716 |
| *TCP11* | 372.7318 | 132.0035 | 44.94669 | 380.3189 | 392.3875 | 320.2378 | 1208.912 | 1015.693 | 874.8646 |
| *ADAM18* | 6.879501 | 1.330068 | 0.975998 | 7.241691 | 12.25686 | 19.5102 | 13.95525 | 14.28711 | 22.95063 |
| *SPA17* | 219.525 | 57.35385 | 30.67272 | 128.8364 | 131.9205 | 193.0506 | 340.1473 | 272.7582 | 381.7373 |
| *SPAG5* | 72.57648 | 49.23824 | 29.98228 | 108.2697 | 163.0371 | 126.8377 | 145.7225 | 173.4632 | 165.5507 |
| *TCTE3* | 41.68199 | 8.99273 | 3.627057 | 14.35482 | 29.0616 | 32.9838 | 44.23411 | 42.67081 | 51.74756 |
| *TCTE1* | 18.05342 | 8.754304 | 2.261969 | 32.70918 | 53.67389 | 40.47088 | 82.2574 | 91.33319 | 72.11014 |
| *TESK1* | 14.50648 | 15.66066 | 9.55554 | 50.64113 | 46.98032 | 36.9915 | 67.91626 | 99.83494 | 60.73692 |
| *CATSPER1* | 20.48492 | 9.246705 | 1.780948 | 23.09255 | 16.51646 | 8.93127 | 75.37422 | 106.727 | 82.09107 |
| *GSG1* | 185.9178 | 79.72231 | 19.37183 | 157.2687 | 72.55718 | 50.64272 | 686.7664 | 497.1728 | 341.6308 |
| *PRM1* | 4396.271 | 2398.097 | 812.9659 | 5579.117 | 2472.185 | 1684.571 | 31635.58 | 17031.92 | 9932.877 |
| *TNP2* | 3.098828 | 2.36391 | 0.631926 | 18.13317 | 18.42958 | 7.486176 | 96.88238 | 119.1923 | 42.85576 |
| *PLCZ1* | 0 | 0 | 0 | 0 | 0 | 0 | 0 | 0 | 0 |
| *TEX264* | 3.907806 | 2.541436 | 1.452022 | 8.47128 | 15.73629 | 12.00992 | 2.573594 | 4.357678 | 3.382929 |
| *GFRA1* | 5.526096 | 2.406266 | 5.129261 | 0.843514 | 0.403724 | 0.777324 | 1.315046 | 1.086555 | 0.643434 |

Supplementary Table 3 the information of included OA patients

| Identifier | Age | LTV^a^ (ml) | RTV^b^ (ml) | Palpation | Utralsonic Inspection | Sperm Obtained via Testicular Biopsy | Tissue Uses |
| --- | --- | --- | --- | --- | --- | --- | --- |
| P3736 | 22 | 12 | 12 | Bilateral epididymal tubercle | tubular ectasia of epididymis bilaterally, with right caput epididymis calcifications | Yes | RNA-Seq |
| P3746 | 23 | 13.5 | 17.4 | Bilateral epididymal tubercle | thin netlike ectasia of corpus and cauda epididymis bilaterally | Yes | RNA-Seq |
| P3750 | 31 | 13 | 13.4 | Bilateral epididymal tubercle | thin netlike ectasia of corpus and cauda epididymis bilaterally | Yes | RNA-Seq |
| P3755 | 25 | 14.1 | 14.7 | Bilateral epididymal tubercle | bilateral epididymal echo-texture inhomogeneity | Yes | RNA-Seq |
| P3790 | 27 | 10.7 | 10.6 | Bilateral epididymal tubercle | thin netlike ectasia of corpus and cauda epididymis bilaterally | Yes | RNA-Seq |
| P3901 | 28 | 10.1 | 12.7 | Bilateral epididymal tubercle | N/A | Yes | RNA-Seq |
| P3834 | 28 | 15 | 15 | N/A* | N/A | Yes | RNA-Seq |
| P3795 | 32 | N/A | N/A | N/A | thin netlike ectasia of epididymis bilaterally | Yes | RNA-Seq |
| P6091 | 27 | N/A | N/A | Bilateral epididymal tubercle | N/A | Yes | RNA-Seq |
| P6095 | 38 | 15.1 | 14.2 | Bilateral epididymal tubercle | thin netlike ectasia of epididymis bilaterally | Yes | RNA-Seq |
| P6090 | 25 | 13.8 | 14.2 | Bilateral epididymal tubercle | thin netlike ectasia of corpus and cauda epididymis bilaterally | Yes | RNA-Seq |
| P3838 | 26 | 12.4 | 13.4 | Bilateral epididymal tubercle | N/A | Yes | RNA-Seq |
| P6100 | 24 | 15.3 | 15.1 | Bilateral epididymal tubercle | N/A | Yes | RNA-Seq |
| P3789 | 30 | 11 | 11.8 | Bilateral epididymal tubercle | N/A | Yes | RNA-Seq |
| P3221 | 29 | 16.8 | 18.4 | Bilateral epididymal tubercle | thin netlike ectasia of caput epididymis bilaterally | Yes | RNA-Seq |
| P3792 | 24 | 15.5 | 15.5 | Bilateral epididymal tubercle | thin netlike ectasia of epididymis bilaterally | Yes | Results Verification |
| P3742 | 28 | 16.1 | 15.8 | Bilateral epididymal tubercle | thin netlike ectasia of corpus and cauda epididymis bilaterally | Yes | Results Verification |
| P3739 | 31 | 15 | 15 | Bilateral epididymal tubercle | thin netlike ectasia of corpus and cauda epididymis bilaterally | Yes | Results Verification |
| P3719 | 38 | 15 | 15 | Bilateral epididymal tubercle | N/A | Yes | Results Verification |
| P3702 | 29 | 15 | 15 | Bilateral epididymal tubercle | thin netlike ectasia of epididymis bilaterally | Yes | Results Verification |
| P3716 | 44 | 16.8 | 14 | Bilateral epididymal tubercle | thin netlike ectasia of epididymis bilaterally | Yes | Results Verification |
| P3712 | 37 | 15 | 15 | Bilateral epididymal tubercle | N/A | Yes | Results Verification |
| P3600 | 21 | N/A | N/A | Bilateral epididymal tubercle | N/A | Yes | Results Verification |
| P3772 | 34 | 10.5 | 9.8 | Bilateral epididymal tubercle | hygroma of right epididymis，left corpus epididymis low echo-texture with calcifications | Yes | Results Verification |
| P3682 | 29 | 10 | 12.4 | Bilateral epididymal tubercle | thin netlike ectasia of corpus and cauda epididymis bilaterally | Yes | Results Verification |
| P3782 | 45 | 13.49 | 13.49 | Bilateral epididymal tubercle | thin netlike ectasia of corpus and cauda epididymis bilaterally | Yes | Results Verification |
| P3721 | 29 | 16.8 | 18.4 | Bilateral epididymal tubercle | thin netlike ectasia of caput epididymis bilaterally | Yes | Results Verification |

* This patient had accepted vasoligation 2 years ago.

a, LTV=Left Testicular Volume

b, RTV=Right Testicular Volume

Supplementary Table 4 Primers used performed Q-PCR

| Gene | Forward Primer | Reverse Primer | Produt Size (bp) |
| --- | --- | --- | --- |
| *CAPN2* | CCGAGGAGGTTGAAAGTAAC | TCCTCTGGGTCTATAGTGTTC | 127 |
| *MPZL1* | CTTGACAGCTGGAGTATCAG | CGTACTAGTAGACTTGAACTTGC | 106 |
| *PRDX1* | GCACCATTGCTCAGGATTAT | CCAACAGGGAGGTCATTTAC | 121 |
| *ITGB1* | GCTCAAGCCAGAGGATATTAC | ACAGGTCCATAAGGTAGTAGAG | 140 |
| *PRDX3* | TTCCCACTTTAGCCATCTTG | CTTCTAACAGCACACCGTAG | 128 |
| *TRIM66* | CCCTCTGTGCTACTTACTCT | GCAGAACTCACGATCCATAC | 136 |
| *ITGA5* | CAGATGCCACACAAGGATAG | CAATGTCTGAGTCTGGGTTC | 127 |
| *LDHB* | GGATATACCAACTGGGCTATTG | GCTCAGGAAGACTTCATTCTC | 135 |
| *MLL2* | CTCCTTTAGTGGAATGAGTGG | CTGGTGGTAACGGAACTTATAG | 121 |
| *WSB2* | GGTCTTTCTATGGAGCATGAG | GTATCGTAAGAAGCCGTGAC | 123 |
| *B2M* | GCAAGGACTGGTCTTTCTATC | CATGTCTCGATCCCACTTAAC | 128 |
| *CD276* | GATCAAACAGAGCTGTGAGG | GGCTATTTCTTGTCCATCATCT | 130 |
| *GOLGA8DP* | GATGTAGGGAGATATGTATGTGTG | CTGCTGCTCTCCTGTTAATG | 128 |
| *ZNF609* | GTTCCTTAGACCCTGGAATAAC | CATTGGGAGACTGCATTTCTA | 127 |
| *C16orf96* | GAAGAGGGCTGTCAAGTATTC | CCTCCCATATTGGCTAGATAAC | 132 |
| *MTSS1L* | TACTGTTCTCTCCAGTGTCTC | CAGAGAGTCAAAGCCAATCTT | 131 |
| *GALNT1* | AACCAGTCGTCATTCCTAAAG | CACCCTTCTAACCTAACATCTG | 130 |
| *ARHGAP33* | CACGAGTTTGACAGTGAGAG | ACTTCCCATAGAGCTGGTAG | 139 |
| *GRIK5* | TTACAAGTACATCCTCACCAC | GGACAAACTCAGGGTAGAAG | 128 |
| *PPP1R15A* | CTGCTACAGGTGTCTTCTTG | GTCTCAGCTTCTCTTTCATCC | 115 |
| *RFX1* | GACTATTGTGCGATTCGTGAG | GCCATAAACATCTATCTCACAGG | 129 |
| *ZNF628* | GAGAGGGAATCGGGATTAATAAG | GAGTTGTAGTTCTCCCAGTTG | 140 |
| *ATIC* | CTTCTTCCCTTTCCGAGATAAC | CGTATGAGCGAGGATGATTC | 136 |
| *HSPD1* | GTCCATTGTACCTGCTCTTG | GAAGACCAACCTTTAGCCTATT | 122 |
| *LRRFIP1* | CTCTCTAGCAGAAGTTGAAGAG | TCAAGCTCCAGCAACATATC | 123 |
| *YWHAB* | AACAGAGAGGAATGAGAAGAAG | GTTGTGTAGCATTGGGAATAAG | 131 |
| *YWHAH* | CTCAACTTCTCCGTGTTCTAC | GTCCTTATAGGAATCCTCGTTTAG | 126 |
| *NFKBIZ* | CGCTTATGAACCAAACCTCT | GGAAGTCTGCATGGGATTAG | 121 |
| *TMEM43* | TGTGTCCATAGCTTTAGTCTTC | AGACATCCTGGTTACAACATAC | 121 |
| *UGDH* | ACTGAGTGGGACATGTTTAAG | GTTTCAATCTGGAAGCCAATG | 140 |
| *HSPA9* | CTCCATTTATCCGCCATGAT | CTCATGACTAAGGCCATTCC | 123 |
| *VCAN* | TCAGGACTACAAAGGGAGAG | GTGTCTTCAATCCCGTACAT | 135 |
| *ID4* | TTTCAGTAGGAAGCATGTCAC | GTTCACAGTCCACCAGTATAAG | 123 |
| *AEBP1* | GATGGAGTCACACCGTATTG | CACGCACCATCATAGTAGTC | 132 |
| *AKR1B1* | CTCCAACTTCAACCATCTCC | GACTGGCAGTACTGGATTAAC | 129 |
| *CAPZA2* | GATGAAGAGAAGGTGCGTATAG | CTTCCCTGAGAAGATTGTCATT | 118 |
| *FAM3C* | ATGTTGGAAGAGGGATCAATG | CCATCTTGTATGGCCTTCAG | 130 |
| *C8orf33* | ACATGCCTGATGAAGAGTTTAG | GACAACAGAAGTCCTGGAAAG | 127 |
| *BEND2* | CATATCCCAGAATTGTCTCCTC | GAGATGACAGCATTGGTACTT | 127 |
| *PHF16* | CAGGCAGAAACTTGGAGAAG | TCCACTCGTACCAAGGAATAG | 138 |
| *RBM3* | GTGTGTCTTGGGTGGTAAAG | CTAGAACCAATGGAGGGATAAC | 135 |
| *HOXB4* | GACAGAAAGAGAAATAGGAGGAG | GCAGAGGAAACAAGACAGAT | 117 |
| *HOXB5* | TCTCTCCTGGACTGGTTATC | CAACCACAGACACAAACATTC | 131 |
| *HOXB8* | TCCCTATCTGACTCGTAAGC | GGAACTTGTCTTTGTTGTTCTC | 128 |
| *HOXC5* | CACTTTAACCGCTACCTCAC | ATCTTTCTTCCACTTCATCCTG | 117 |
| *HOXC6* | AAAGCCAGTATCCAGATTTACC | AGTGAAATTCCTTCTCCAGTTC | 136 |
| *HOXC8* | GAGACGCCTCCAAATTCTATG | CCTTCGCTACTGTTAGTGTTG | 127 |
| *HOXC11* | TGTTTAACTCGGTCAACCTG | GAACTCGGGCATGTAGTAAG | 131 |

Supplementary Figures


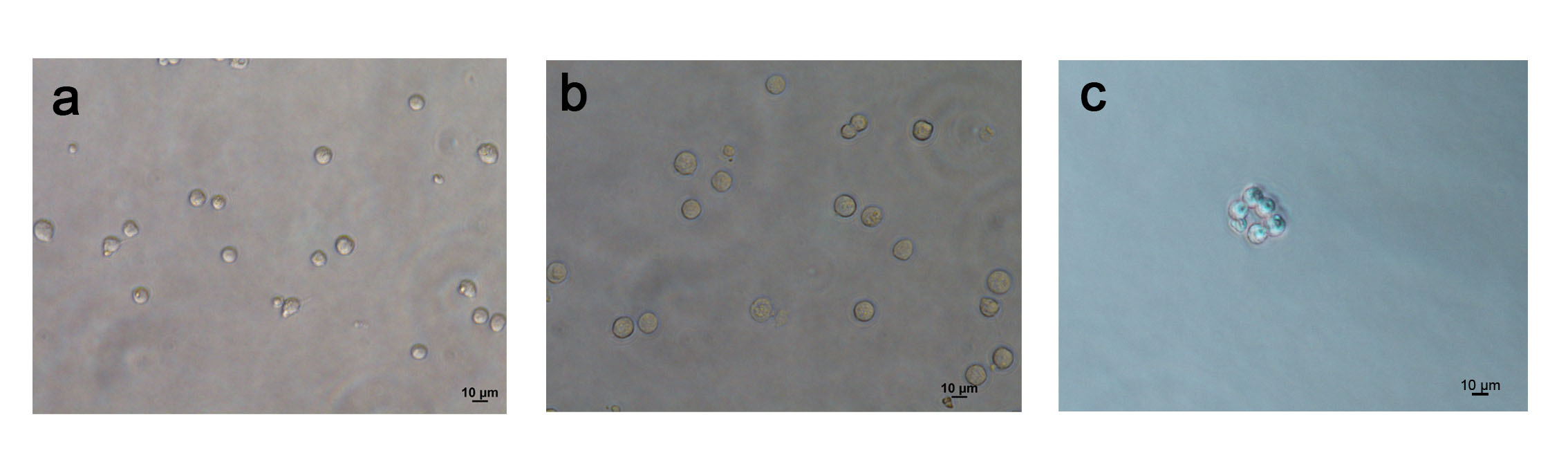


Supplementary Figure 1 Morphology of sorted celles

A) Sorted haploid cells. The diameter of most cells is less than 10μm, which matches the size feature of spermatid. Elongated spermatid with a short tail could also be observed. B) Sorted tetrapolid cells. The diameter of most cells is above 15 μm, which matches the size feature of primary spermatocyte. C) Sorted CD90+ cells. The diameter of these cells is about 10 μm, which matches the size feature of spermatogonial cell.


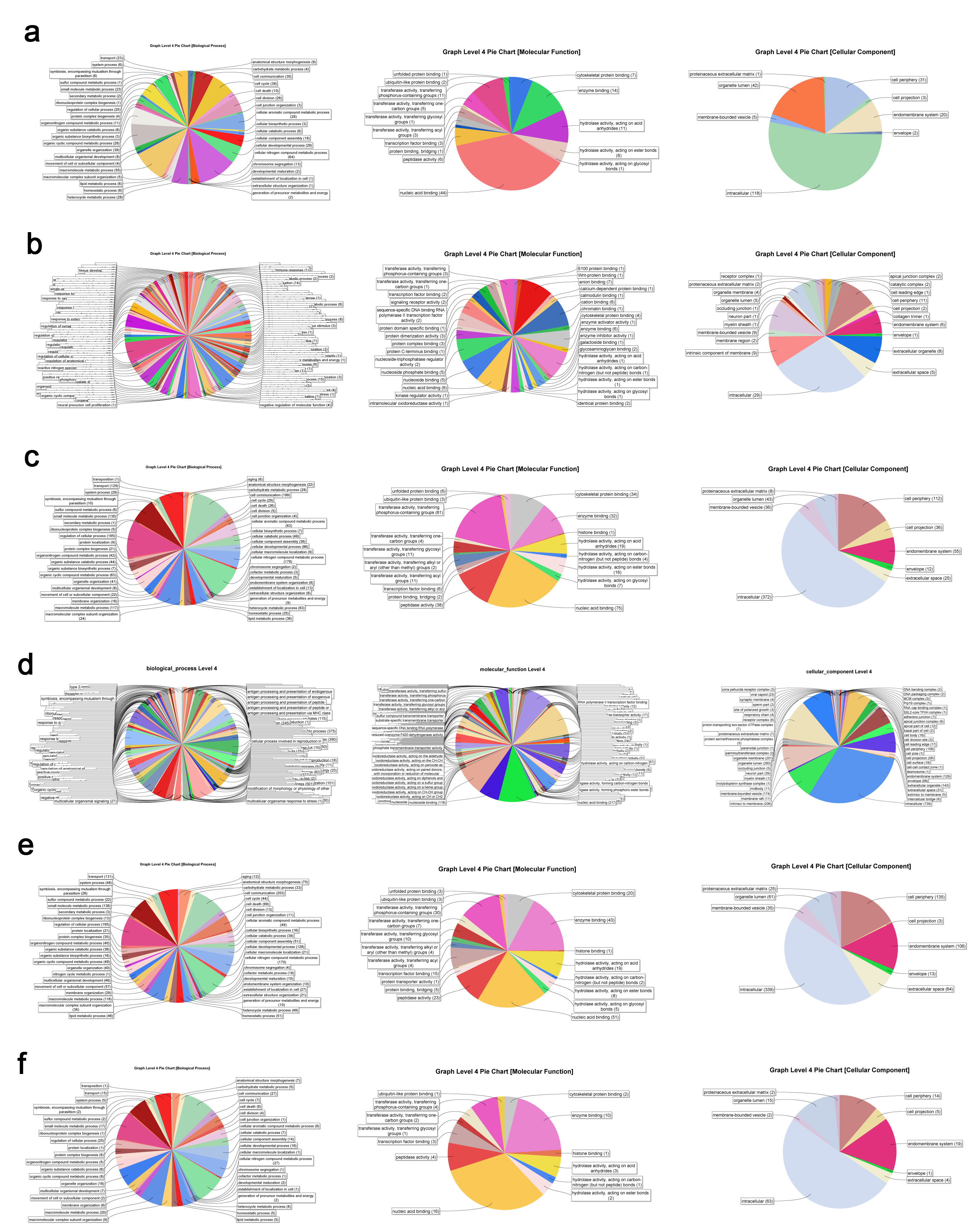


Supplementary Figure 2 Pie charts of GO annotation for the differential expressed genes from Cluster 5-10

A) Pie chart of GO annotation for the differential expressed genes from Cluster 5, which were preferentially up-regulated in spermatocytes; B) Pie chart of GO annotation for the differential expressed genes from Cluster 6, which were preferentially down-regulated in spermatocytes; C) Pie chart of GO annotation for the differential expressed genes from Cluster 7, which were preferentially up-regulated in spermatids; D) Pie chart of GO annotation for the differential expressed genes from Cluster 8, which were preferentially down-regulated in spermatids; E) Pie chart of GO annotation for the differential expressed genes from Cluster 9, which were preferentially up-regulated in undifferentiated spermatogonias; F) Pie chart of GO annotation for the differential expressed genes from Cluster 10, which were preferentially down-regulated in undifferentiated spermatogonias
